# Greener Is Better: First Approach for the Use of Natural Deep Eutectic Solvents (NADES) to Extract Antioxidants from the Medicinal Halophyte *Polygonum maritimum* L.

**DOI:** 10.3390/molecules26206136

**Published:** 2021-10-11

**Authors:** Iva Rukavina, Maria João Rodrigues, Catarina G. Pereira, Inês Mansinhos, Anabela Romano, Sylwester Ślusarczyk, Adam Matkowski, Luísa Custódio

**Affiliations:** 1 Centro de Ciências do Mar (CCMAR), Campus de Gambelas, Universidade do Algarve, Ed. 7, 8005-139 Faro, Portugal; Iva.Rukavina@imbrsea.eu (I.R.); mjrodrigues@ualg.pt (M.J.R.); cagpereira@ualg.pt (C.G.P.); 2 Campus de Gambelas, MED-Mediterranean Institute for Agriculture, Environment and Development, Faculdade de Ciências e Tecnologia, Universidade do Algarve, Ed. 8, 8005-139 Faro, Portugal; ifmansinhos@ualg.pt (I.M.); aromano@ualg.pt (A.R.); 3 Department of Pharmaceutical Biology and Biotechnology, Botanical Garden of Medicinal Plants, Wroclaw Medical University, 50-556 Wroclaw, Poland; sylwester.slusarczyk@umed.wroc.pl (S.Ś.); pharmaceutical.biology@wp.eu (A.M.)

**Keywords:** NADES, green chemistry, antioxidant extraction, halophytes

## Abstract

In this study, natural deep eutectic solvents (NADES) formed by choline chloride (ChCl), sucrose, fructose, glucose, and xylose, were used to extract antioxidants from the halophyte *Polygonum maritimum* L. (sea knotgrass) and compared with conventional solvents (ethanol and acetone). NADES and conventional extracts were made by an ultrasound-assisted procedure and evaluated for in vitro antioxidant properties by the radical scavenging activity (RSA) on the 2,2-diphenyl-1-picrylhydrazyl (DPPH) radical, oxygen radical absorbance capacity (ORAC), and copper chelating activity (CCA). Samples were profiled by liquid chromatography (LC)-electrospray ionization (ESI)-QTOF-MS analysis. ChCl:fructose was more efficient in the DPPH assay, than the acetone extract. ChCl:sucrose and ChCl:fructose extracts had the highest ORAC when compared with the acetone extract. NADES extracts had higher CCA, than the acetone extract. The phenolic composition of the NADES extracts was less complex than the conventional extracts, but the proportions of major antioxidants, such as flavonols and flavan-3-ols, were similar in all the solvents. Myricitrin was the major flavonoid in all of the samples, while gallic acid was the main phenolic acid in the conventional extracts and present in a greater amount in ChCl:fructose. Results suggest that NADES containing ChCl and sucrose/fructose can replace conventional solvents, especially acetone, in the extraction of antioxidants from sea knotgrass.

## 1. Introduction

Organic solvents such as methanol, ethanol, and acetone are commonly used in the conventional extraction of natural compounds, due to their high extraction and separation capacities [1]. However, their use has several disadvantages, including high solvent consumption, long extraction times, and low reuse potential, leading to a consequently higher price of the extraction process [2]. Moreover, these organic solvents have a negative impact on the environment, due to their toxicity, volatility, solubility, and flammability [3]. Green chemistry had tackled the pollution problem with the identification of environmentally friendly solvents and/or separation processes and has the legislative support of the EU environmental policy [4,5]. One of the possible suggested green solvents are, for example, ionic liquids (ILs), which are liquid salts at room temperature made of different cations (e.g., alkyl-imidazolium, pyridinium, ammonium, phosphonium) and inorganic anions (e.g., acetate, trifluoroacetate, trifluoromethyl sulfate) [6]. When compared with conventional solvents, ILs have a higher solubility, tuneability, and stability, a lower melting point and vapor pressure, and no volatility and flammability. Nevertheless, ILs still exhibit low biodegradability, high costs, and some constituents are toxic [4,7,8]. Deep eutectic solvents (DES) are novel liquid salts. In addition, their principle is that a hydrogen bond acceptor (HBA), generally quaternary ammonium salts such as choline chloride (ChCl), combined with different hydrogen bond donors (HBD), including amines, alcohols, carboxylic acids, sugars, and vitamins, in a specific molar ratio, result in a mixture with a relative melting point lower than their individual components [7,9]. ILs and DES have similar physicochemical properties, but DES are easier and more affordable to synthesize and store [8]. Moreover, DES can be tailored to produce combinations with suitable physicochemical features that allow for the extraction of a specific group of bioactive molecules, for a particular purpose [10].

A few molecules, including sugars and choline, are present in high amounts in microbes, mammals, and plants cells and show the same properties as DES. In other words, they change their state from solid to liquid when mixed in a proper ratio [11,12]. The hypothesis is that the mixture of the primary metabolites forms an alternative media to water and lipids in the living organisms, which enables the synthesis of poorly water-soluble or insoluble molecules (e.g., rutin, cellulose, and lignin), and biochemical reactions in harsh environmental conditions such as drought, cold, and salt stress [10]. This concept led to the discovery of natural deep eutectic solvents (NADES), a new generation of DES, consisting of a mixture of primary metabolites such as sugars, organic acid, amino acids, and chlorine derivates [3]. NADES have several advantages over conventional extraction and ILs, including lower cost, biodegradability, and reduced toxicity [3,4,7,8]. NADES have been applied for the extraction of several bioactive natural products from marine and terrestrial sources, with high potential future use in commercial purposes, including hydrophilic and lipophilic compounds from *Fucus vesiculosus* [13], phlorotannins from brown algae [14], anthocyanins from blueberry peels [15] and gray berry [16], anthocyanins and phenolics from grape (*Vitis vinifera*) [7,9,17], flavonoids from Japanese pagoda tree (*Sophora japonica*), onion (*Allium cepa*), broccoli (*Brassica oleracea var. italica*), cranberry (*Vaccinium* sp.), plum (*Prunus* sp.), rosemary (*Salvia rosmarinus*), and pepper (*Piper nigrum*) [18,19,20], phenolic acids from *Lonicerae japonicae* [1], polyphenols from dittany (*Origanum dictamnus*), marjoram (*Origanum majorana*), mint (*Mentha spicata*), sage (*Salvia officinalis*), and fennel (*Foeniculum vulgare*) [21], phenylethanes and phenylpropanoids from *Rhodiola rosea* L. [22], and steroidal saponins from *Dioscoreae nipponicae* rhizome [23].

*Polygonum maritimum* L. (sea knotgrass) is a Mediterranean-Atlantic halophyte species that occurs in Mediterranean and Black Sea coasts and reaches its northern limits in the Channel Islands, England, and Belgium. Ethanol and acetone extracts from sea knotgrass display multiple biological properties, including in vitro anti-diabetic, anti-inflammatory, anti-aging, anti-bacterial, and anti-fungal [24,25,26,27,28]. The phytochemical analysis of the extracts made from leaves and roots allowed the identification of several bioactive compounds belonging to different classes, such as tannins, saponins, flavonoids, terpenoids, cardiac glycosides, fatty acids, phenols, alcohols, and phytosterols [25,26]. Therefore, sea knotgrass has a promising medical, cosmetic, and nutritional application, and can be cultivated in saline conditions while maintaining its biochemical properties [29]. In this work, we evaluated the efficiency of NADES as a possible green replacement for conventional solvents in the extraction of antioxidant compounds from sea knotgrass, in the context of the sustainable exploitation of this species as a source of bioactive products to be applied in the food industry, as antioxidant food additives.

## 2. Results and Discussion

### 2.1. Preparation and Optimization of NADES

In this work, four NADES were prepared containing choline chloride (ChCl) as a hydrogen bond acceptor (HBA) and glucose (Gluc), fructose (Fruc), xylose (Xyl), and sucrose (Suc) as hydrogen bond donors (HBD) (Table 1). These NADES fit into the green chemistry concept and the green extraction approach for the design of products to be profitable, while safe for human health and the environment [1,4,30,31]. Choline is inexpensive, non-toxic, biodegradable, and commonly used as a vitamin for animal feed [1,32]. In addition, it is provided by several foods, including eggs and dried soybeans [33], and is an essential element for several cellular processes, including the synthesis of the neurotransmitter acetylcholine and cell membranes phospholipids [34,35]. ChCl is synthesized from a trimethylamine, ethylene oxide, and hydrochloric acid (HCl) in a reaction that has the E (environmental) Factor close to zero [36]. The E factor is used to assess the environmental impact of industrial production by calculating the kilograms of waste generated for each kilogram of the produced product [37]. The HBDs used in this study were the naturally occurring sugars Gluc, Fru, and Suc, which are used as sources of energy for living organisms, in food or sweeteners, with low toxicity concerns [38,39]. Xylose or wood sugar naturally occurs in hardwoods and agricultural residues [40] and builds cell walls of the cereal grains [30]. Xylose is poorly metabolized by monogastric animals including humans [30]. The low or nil toxicity of ChCl based NADES with Gluc, Fru, Suc, and Xyl was previously confirmed on fish (CCO) and human (MCF-7, HeLa, L929) cell lines, by inhibition of bacterial growth (*Escherichia coli*, *Staphylococcus aureus*, Salmonella enteritidis, and *Listeria moncytogenes*) and by phytotoxicity assays on the germination and early growth of wheat (*Triticum aestivum*) [3,18,41,42].

The ultrasound-assisted NADES synthesis method used in this work was more efficient in terms of time and energy saving, when compared to previously published results for the commonly used heating method of synthesis. The ultrasound-assisted synthesis took 15–60 min at the temperature of 50 or 55 °C (Table 1), while in the literature the heating method of synthesis for the same HBA:HBD mixture, although in some cases with different molar ratios, lasted from 1–6 h at 80 °C [1,41].

When mixing the NADES components, a clear, transparent, and homogeneous liquid was formed. However, a crystalline precipitate was occasionally observed. Therefore, NADES were considered stable if no significant crystallization became visible after 7 days of storage, in the dark, at room temperature (approx. 20 °C). The combinations ChCl:Glu and ChCl:Xyl exhibited crystallization after 2 to 3 days, and therefore, were not selected for further analysis. Dai et al. [43] studied different ratios of NADES components in terms of stability and reported that ChCl:Gluc, at a molar ratio of 1:2, also precipitate within 7 days. The same authors observed that ChCl:Xyl at different molar ratios from those used in this work (2:1 and 3:1) were stable. At certain molar ratios, the hydroxyl or carboxyl groups from HBA, in this case ChCl, cannot combine with hydroxyl groups from HBD, Gluc, and Xyl, due to the unequal number of hydrogen bond donor and acceptor groups, resulting in the formation of a solid precipitate [43,44].

### 2.2. In Vitro Antioxidant and Metal Chelating Properties

**Figure 1:**
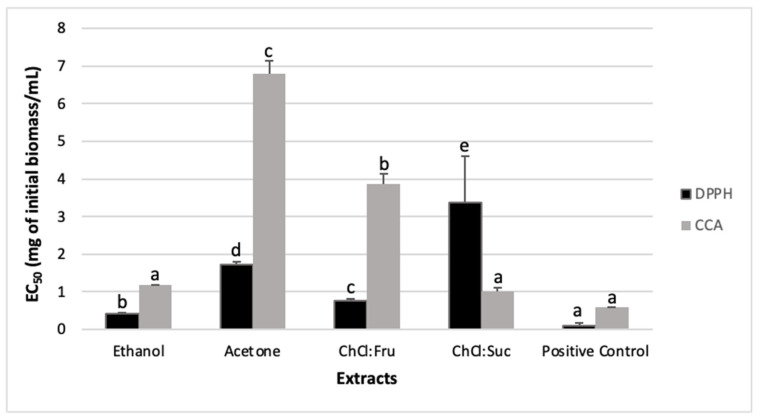
Radical scavenging activity (RSA) on 2,2-diphenyl-1-picrylhydrazyl (DPPH) and copper chelating activity (CCA), expressed as EC_50_ values (mg of initial biomass per mL) of the extracts from *Polygonum maritimum* L. (sea knotgrass). Results are presented as mean values ± standard error (SE) of at least six replicates (n = 6). Columns with different letters are significantly different at *p* < 0.05 (Duncan’s new multiple range test). Positive control for DPPH: BHT (butylated hydroxytoluene); positive control for CCA: EDTA (ethylenediaminetetraacetic acid).

It is essential to evaluate the antioxidant activity of the plant extracts with more than one assay, due to their high mixtures’ complexity. In this work, the antioxidant activity was evaluated using three different in vitro chemical assays. The radical scavenging activity (RSA) towards the 2,2-diphenyl-1-picrylhydrazyl (DPPH) radical estimates the capacity of the tested sample to scavenge free radicals [45]. In addition, it is a standard, simple, fast, and widely used method for measuring the in vitro antioxidant activity of natural extracts [46]. In this work, the highest RSA towards the DPPH radical was obtained for the ethanol extract (half maximal effective concentration—EC_50_ value = 0.421 mg/mL), followed by ChCl:Fru (EC_50_ = 0.773 mg/mL), acetone (EC_50_ = 1.725 mg/mL), and ChCl:Suc (EC_50_ = 3.373 mg/mL) (Figure 1). When evaluating the DPPH RSA of acetone extracts from sea knotgrass cultivated in a greenhouse under different salinity irrigation conditions, Rodrigues et al. [29] found EC_50_ values in the range of 138–679 µg/mL, depending on the salinity and harvest, which are lower than the values observed in this work.

**Figure 2:**
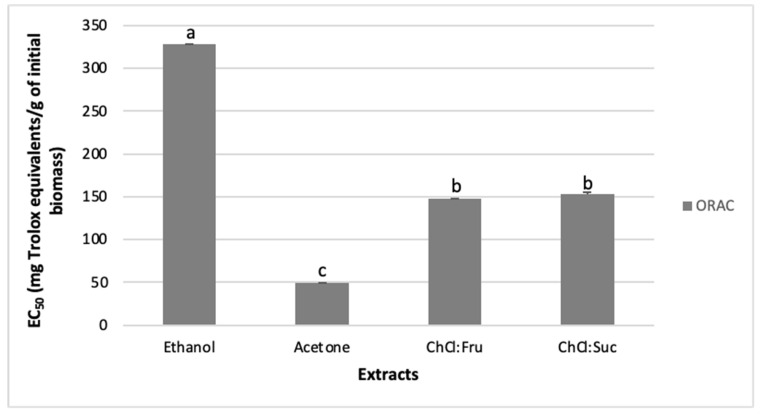
Oxygen radical absorbance capacity (ORAC) values (expressed as mg of Trolox equivalent (TE) per g of initial biomass) of tested extracts. Results are presented as mean values ± standard error (SE) of at least six replicates (n = 6). Columns with different letters are significantly different at *p* < 0.05 (Duncan’s new multiple range test).

The oxygen radical absorbance capacity (ORAC) assay measures the capacity of the tested sample to inhibit the oxidation of the peroxyl-radical [47]. It is considered as a biologically relevant method, since peroxyl radicals naturally occur in the living organisms, which are implicated in the propagation step of lipid peroxidation that is linked to the pathogenesis of several diseases, including atherosclerosis and asthma [41,48,49]. In the ORAC assay, the ethanol extract (328 mg trolox equivalentes (TE)/g) was the more efficient extract, followed by ChCl:Suc (153.51 mg TE/g), ChCl:Fru (148.12 mg TE/g), and acetone (49.34 mg TE/g) (Figure 2).

Methods targeting the chelating properties towards redox metals evaluate the capacity of the samples to form stable chelates with metal ions and complement the radical based methods, since excess free metals contribute to formation of the free radicals, and therefore, for the occurrence of oxidative stress [50]. Moreover, the accumulation of redox metals in the organism, such as copper, is implicated in the etiopathogenesis of several human diseases, such as Wilson’s syndrome, Alzheimer’s and Parkinson’s diseases, and in several cancer types [51]. The extracts with the higher capacity to chelate copper were ChCl:Suc (EC_50_ = 1.008 mg/mL) and ethanol extracts (EC_50_ = 1.169 mg/mL), followed by ChCl:Fru (EC_50_ = 3.868 mg/mL) and acetone extracts (EC_50_ = 6.783 mg/mL) (Figure 1). The copper chelating activity (CCA) of the ChCl:Suc and ethanol extracts were significantly equivalent to the used positive control (ethylenediaminetetraacetic acid (EDTA), EC_50_ = 0.579 mg/mL) (Figure 1).

When comparing the antioxidant activity of NADES and conventional extracts, it was observed that the ethanol extracts exhibited higher antioxidant properties, except in the CCA, where its activity was comparable to ChCl:Suc (Figure 2). The mixture ChCl:Fru always exhibited stronger antioxidant properties than the acetone extract (Figure 2). These results suggest that NADES, specifically ChCl:Suc and ChCl:Fru mixtures, could replace acetone and ethanol in the extraction of antioxidant compounds from sea knotgrass. These results follow other reports where the antioxidant activity of NADES extracts was generally higher or similar than those of extracts using conventional solvents, such as ethanol and methanol [21,41,45,52,53]. For example, Pal and Jadeja [52] observed that NADES extracts from onion peel made with ChCl and Suc, urea, and sorbitol had significantly (2–5 times) higher capacity to reduce iron than the aqueous methanol extract. In the same study, the DPPH RSA of ChCl:sorbitol was similar to the methanol extract [52]. In another study, the ORAC values for grape skin extracts made with ChCl and different sugars, including Gluc, Fru, and Xyl, were higher than those obtained with aqueous methanol extracts [41]. The DPPH RSA properties of *Curcuma longa* NADES extracts were higher than those obtained with the methanol extract, but lower when compared with the ethanol one [44]. These results were related to different extraction yields and different extracted chemical components [41,44]. All in all, the extraction efficiency of target functional molecules and the antioxidant activity suggest NADES as a sustainable alternative for the use of conventional solvents in the extraction of bioactive components from different plant species [21,41,44,52,53].

### 2.3. Phytochemical Characterization of Extracts

**Figure 3:**
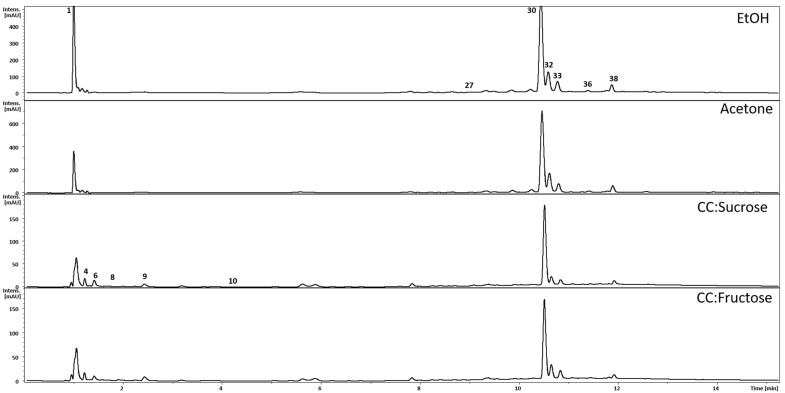
Examples of average HPLC chromatograms of *Polygonum maritimum* L. (sea knotgrass) extracts (UV 254 nm). The major peaks are numbered according to Table 2. The relative signal intensities shown on the X-axes are significantly stronger in both acetone and ethanol extracts than in NADES.

**Figure 4:**
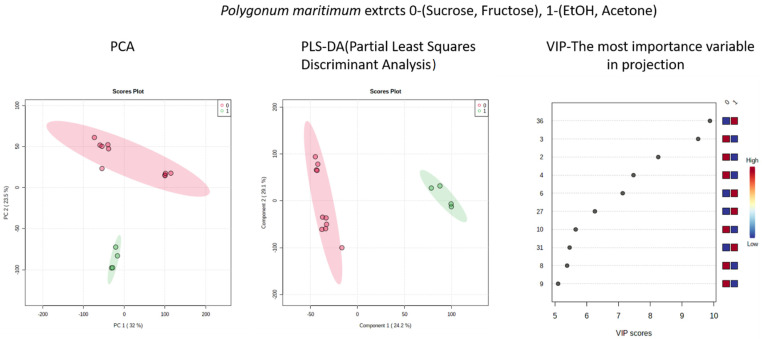
Multivariate analysis (principal component analysis (PCA) and partial least square discriminant analysis (PLS-DA) of phytochemical (LC-MS) data showing the clustering of samples depending on the solvents. NADES extracts are shown as red circles and ethanol and acetone as green circles. The variable importance in the projection (VIP) chart shows peaks (numbered as in Table 2) that most contribute to the differentiation between the extracts.

Altogether, 51 peaks were detected, of which 43 were annotated (Table 2, Figure 3). Most of the peaks belonged to phenolic compounds representing flavonols, flavan-3-ols, and phenol carboxylic acids. The peaks corresponding to sucrose and fructose are likely to be artifacts. As expected, the NADES extracts were highly loaded with these sugars and the solid phase extraction (SPE) procedure may not be able to completely remove all of them. Moreover, this is the most likely reason for the apparently lower absolute amounts of the major flavonols in the analytical samples. The crude extracts had to be washed off the syrupy solvents and evaporated to dryness. Therefore, the final amounts eluted from the high-performance liquid chromatography (HPLC) column were reduced. However, the relative proportions between the individual flavonols and flavan-3-ols were largely retained (Figure 1). The general composition of the polyphenols regardless of the extraction solvent was similar to our previous studies [27,28,29] except for polygonophenone, the unique compound isolated by Kazantzoglou et al. [54], detected only in the earlier studies by Rodrigues et al. [27,28], and not detected in this study. This may be explained by the different plant biomass used and suggest the influence of environmental factors, rather than the genetic determination of this compound production in sea knotgrass. Conversely, myricitrin remained the main flavonoid compound in all our studies focusing on sea knotgrass [27,28]. The main quantitative difference between the NADES and conventional extracts was in the content of gallic acid, which was a major compound in the ethanol and acetone extracts, but only a minor, unquantifiable peak in NADES (Figure 1 and Table 2). Gallic acid (3,4,5-trihydroxylbenzoic acid) is common in different plant species, such as leafy vegetables and fruits. Gallic acid and its glucosides derivatives exhibit relevant biological properties, including antioxidant [55], which may have accounted, at least in part, to the highest antioxidant activity of the ethanol extract, in the DPPH and ORAC assays. The multivariate analysis [both principal component analysis (PCA) and partial least squares discriminant analysis (PLS-DA)] of the extract’s composition shows clearly that NADES and ethanol and acetone extracts form two well-separated clusters (Figure 4). The variable importance in projection (VIP) analysis (Figure 4) indicated peaks that most contributed to the LC-MS profile differences between the extracts. Quercetin xyloside (peak **36**) and gallic acid (peak **6**) were the important contributors in the acetone extract, whereas citric acid (4) differentiated both NADES extracts. However, the peaks from sucrose (peak **2**) and fructose (peak **3**) which are both components of NADES also remained as most significant, probably due to the incomplete removal of solvent residues during sample preparation.

## 3. Materials and Methods

### 3.1. Chemicals

Acetone and methanol were obtained from Valente and Ribeiro (Lisbon, Portugal). Ethanol (96%) was purchased from “AGA-Álcool e Géneros Alimentares”, S.A. (Lisbon, Portugal). Monobasic potassium phosphate was from Merck (Darmstadt, Germany), while dibasic potassium phosphate, iron (II) chloride (FeCl_2_), ferrozine, ethylenediaminetetraacetic acid (EDTA), pyrocatechol violet (PV), copper sulfate (CuSO_4_), sodium acetate, choline chloride, glucose, fructose, sucrose, and xylose were provided by VWR International (Leuven, Belgium). Panreack AppliChem ITW Reagents (Barcelona, Spain) supplied fluorescein and Acros Organics (Geel, Belgium) provided 2,2′-azobis (2-amidinopropane) dihydrochloride (AAPH) and Trolox. Sigma-Aldrich (Lisbon, Portugal) delivered DPPH and butylated hydroxytoluene (BHT).

### 3.2. Plant Material

The leaves and stems of sea knotgrass were harvested in the south of Portugal in Fuzeta island (N 37°2′33.079″, W 7°44′47.321″) in August of 2017 and identified by Luísa Custódio, according to the morphological characteristics. A voucher herbarium specimen was placed in the XtremeBio laboratory under the number XBH22.1. The plant material was freeze dried for 3 days, reduced to powder using a coffee mill, and stored at −20 °C before analysis.

### 3.3. Conventional Extraction

Dried biomass was mixed with acetone and ethanol in the ratio of 1:40 (*w/v*) [26]. An ultrasound-assisted extraction was performed in an ultrasonic bath (USC-TH, VWR, Portugal), with a capacity of 5.4 L, frequency of 45 kHz, a supply of 230 V, and a tub heater of 400 W, with temperature control made by a LED display.

The obtained extracts were filtered (Whatman No.4) and the solvent was completely removed in a rotary evaporator under reduced pressure and temperature (approximately 40 °C). The crude ethanol extract was dissolved in ethanol at the concentration of 1 mg/mL, while the crude acetone extract was dissolved at the same concentration in methanol. The extracts were stored in the dark at 4 °C, until analysis.

### 3.4. Extraction Using NADES

#### 3.4.1. Preparation of NADES

NADES were prepared according to published protocols, with some modifications [1,20,41]. ChCl was used as an HBA and Gluc, Fruc, Xyl, and Suc were selected as HBD. The components were previously dried in an incubator for 24 h at 60 °C [41]. ChCl was mixed with every HBD at the molar ratio of 1:2 [1]. The mixture was incubated in an ultrasonic bath, as described in Section 3.3, in closed glass flasks from 15 to 60 min at 50 °C for ChCl:Gluc, and 55 °C for ChCl:Fruc, Xyl, and Suc, until homogeneous and colorless liquids were formed [1,20]. Distilled water (30–40%, *w:w*) was added to NADES [1,20,41], leading to less viscous solutions (Table 1). The obtained NADES were kept in the dark at room temperature [21].

#### 3.4.2. Extraction

Dried biomass of sea knotgrass was mixed with NADES in the ratio of 1:40 (*w/v*), and extraction was made in an ultrasonic bath (conditions described in Section 3.3) for 30 min at room temperature [26]. The extracts were then centrifuged (centrifuge Z 200 A, Hermle Labortechnik GmbH, Germany) for 15 min at 5000 g, and the supernatants were collected and used for further analysis. The extracts were kept in the dark at room temperature to avoid crystallization [41].

### 3.5. Determination of In Vitro Antioxidant and Metal Chelating Properties

In all the assays, if not mentioned differently, serial dilutions of the conventional extracts were used, ranging from 1 to 0.00195 mg/mL of the crude extract. For the NADES extracts, concentrations ranged from 25 to 0.0061 mg initial biomass/mL (which is, mg of dried plant biomass mixed with each mL of the solvent used in the extraction). Before the assays, the extracts made with NADES were warmed for 20 min at 60 °C, to reduce the viscosity. If the extract exhibited crystallization, it was incubated in the ultrasonic bath at 55 °C and the frequency of 45 kHz until the dissolution of the crystals. When the crystallization was not reverted, the extract was not used in the assays.

#### 3.5.1. Radical Scavenging Activity (RSA) towards DPPH

The extracts were tested for RSA on the DPPH radical in 96-well microplates, according to the protocol described in Rodrigues et al. [56]. The extracts (22 µL) were mixed with 200 µL of DPPH (120 µM in ethanol) and incubated in the dark for 30 min at room temperature. Butylated hydroxytoluene (BHT) (1–0.031 mg/mL) and the solvent (ethanol, acetone or NADES) used for the extraction were used as positive and negative controls, respectively. The color control consisted of 22 µL of the extracts mixed with 200 µL of the solvent used for the extraction. The absorbance was measured at 492 nm using a microplate reader. RSA was expressed as the percentage of DPPH reduction, calculated in relation to the negative control, and as half maximal effective concentration (EC_50_ values), when possible.

#### 3.5.2. ORAC Assay

The ORAC assay was conducted in 96-well flat bottom black microplates, according to Gillespie et al. [57]. Conventional extracts were diluted at concentrations ranging from 0.05 to 0.00781 mg/mL with phosphate buffer (75 mM, pH 7.0). NADES extracts were diluted at concentrations ranging from 0.05 to 0.0061 mg initial biomass/mL, also with a buffer. Fluorescein (150 µL, 0.2 µM) was added to the well containing either 25 µL of buffer for the blank control, 25 µL of Trolox (positive control, 6.25, 12.5, 25, and 50 µM) or 25 µL of the different concentrations. The reaction mixture was incubated for 10 min at 37 °C. Then, 25 µL of AAPH (150 mM) were added to the reaction mixture. The fluorescence kinetic read was run at 485 nm excitation and 530 nm emission for 90 min (interval time 5 min, 19 cycles) at 37 °C using a microplate reader (Infinite M200, Tecan Switzerland). The relative fluorescence of each well was calculated, and the fluorescence sample curves as well as the blank and Trolox standards curves were plotted. The ORAC activity of the samples was presented as the net area under the curve (Net AUC), which was calculated as the difference between the area under the sample curve and the area under the blank curve. Based on the Net AUC, the best concentration of 0.0625 mg/mL for both ethanol and acetone extracts was obtained (value closer to the middle value of the Trolox standards of AUC). For the NADES extracts, the best concentration was 0.0061 mg initial biomass/mL. The ORAC values of the samples were finally expressed as mg Trolox equivalent (TE) per g of initial biomass using the quadratic regression equation with the known Trolox concentrations and Net AUC.

#### 3.5.3. CCA

The extracts were tested for CCA in 96-well microplates according to Rodrigues et al. [56]. The samples (30 µL) were mixed with 200 µL of sodium acetate buffer (50 mM, pH 6), 100 µL of copper sulfate pentahydrate (CuSO_4_·5H_2_O, 50 µg/mL, in distilled water), and 6 µL of PV (4 mM, in distilled water). EDTA (1–0.03 mg/mL) was used as a positive control, while the corresponding solvent (ethanol, acetone or NADES) was used as the negative control. The color control consisted of 30 µL of the extracts mixed with 306 µL of sodium acetate buffer. The absorbance was measured at 620 nm using a microplate reader (EZ Read 400, Biochrom United Kingdom). CCA was expressed as the percentage of PV elimination from the Cu complex by the sample in relation to the blank.

### 3.6. Chemical Characterization of the Extracts

#### 3.6.1. Sample Preparation

Each extract (8 mg each) was dissolved in 2 mL of ultra-pure water, acidified with 0.2% (*v/v*) formic acid, and purified by SPE using the Oasis HLB 3 cc Vac Cartridge, 60 mg (Waters Corp., Milford, MA). The cartridges were washed with 2 mL 0.5% (*v*/*v*) aqueous methanol to remove carbohydrates, and then washed with 2 mL 80% (*v*/*v*) aqueous methanol to elute phenolics. The phenolic fraction was re-evaporated, weighed accurately, and re-dissolved in 2 mL 80% methanol (acidified with 0.2% formic acid) to get 3 mg/mL concentration. The sample was then centrifuged (23,000× *g*, 5 min) and filtered (0.22 µm) before liquid chromatography (LC)-electrospray ionization (ESI)-QTOF-MS analyses, that were performed in triplicate for three independent samples (stored at −20 °C before analysis for no longer than 3 days).

#### 3.6.2. LC-(ESI)-QTOF-MS Analysis

LC-(ESI)-QTOF-MS estimation of the polyphenol composition of the extracts was carried out on a Thermo Dionex Ultimate 3000 RS (Thermo Fischer Scientific, Waltham, MA, USA) chromatographic system, coupled to a Bruker Compact (Bruker, Billerica, MA, USA) quadrupole time-of flight (QTOF) mass spectrometer, consisting of a binary pump system, sample manager, column manager, and PDA detector. Separations were performed on a Kinetex C18 column (2.1 × 100 mm, 2.6 μm, Phenomenex, Torrance, CA, USA), with mobile phase A consisting of 0.1% (*v*/*v*) formic acid in water and mobile phase B consisting of 0.1% (*v*/*v*) formic acid in acetonitrile. A linear gradient from 7 to 50% phase B in phase A over 20 min was used to separate phenolic compounds. The flow rate was 0.3 mL/min and the column was held at 30 °C. Spectra were acquired in a negative ion mode over a mass range from m/z 100 to 1500 with 5 Hz frequency. Operating parameters of the ESI ion source were as follows: Capillary voltage 3 kV, dry gas flow 6 L/min, dry gas temperature 200 °C, nebulizer pressure 0.7 bar, collision radio frequency 700.0 V, transfer time 100.0 μs, and pre-pulse storage 7.0 μs. Ultrapure nitrogen was used as drying and nebulizer gas, and argon was used as collision gas. Collision energy was set automatically from 15 to 75 eV depending on the m/z of the fragmented ion. Acquired data were calibrated internally with sodium formate and introduced to the ion source at the beginning of each separation via a 20 μL loop. Processing of the spectra was performed with Bruker DataAnalysis.

#### 3.6.3. LC-MS Data Processing and Compounds Annotation

After data acquisition, the raw UPLC-QTOF-MS spectra (negative mode) were pre-processed using the ProfileAnalysis software (version 2.1, Bruker Daltonik GmbH, Bremen, Germany) with the following settings: Advanced bucket generation with a retention time range of 0−20 min, a mass range of 100–800 m/z, each bucket (spectral bins) was formed with 1 min and 1 m/z delta, 0.2 kernelizing value, without normalization, background subtraction, and time alignment. LC-MS analyses were processed with the find molecular features (FMF) function to create compounds (molecular features) with S/N- 3 for peak detection.

The DataAnalysis 4.3 software (Bruker Daltonics GmbH, Bremen, Germany) provides a ranking according to the best fit of measured and theoretical isotopic patterns, within a specific mass accuracy window. The quality of the isotopic fit was expressed by the mSigma-value. The peaks were matched using the SmartFormula3D function and sent to the MetFrag in silico fragmentation website for computer-assisted identification of the mass spectra, using the Bruker-Sumner MetaboBase^®^ Plant Library (Bruker Corporation, Billerica, MA, USA) [56]. In addition, other databases were used to manually search for the structural identity of the metabolites and these included: HMDB (http://www.hmdb.ca/, accessed on 1 May 2021), Mass Bank of North America (MoNA) (https://mona.fiehnlab.ucdavis.edu, accessed on 1 May 2021), BiGG (http://bigg.ucsd.edu/, accessed on 1 May 2021), PubChem (http://pubchem.ncbi.nlm.nih.gov/, accessed on 1 May 2021), MassBank database (http://www.massbank.jp, accessed on 1 May 2021), KEGG (www.genome.jp, accessed on 1 May 2021), and the Metlin database (http://metlin.scripps.edu, accessed on 1 May 2021).

The annotated compounds were estimated quantitatively using isoquercitrin (CAS 482-35-9 quercetin 3-O-glucopyranoside, Merck, Darmstadt, Germany) as a reference standard for flavonols (compounds marked with superscript F in Table 2) and (-)-epicatechin (CAS 490-46-0, Supelco, Bellefonte, PA, USA) was used for other phenolics (compounds marked with superscript E in Table 2). Stock solutions of (-)-epicatechin and isoquercitrin were prepared in methanol at concentrations of 3.2 and 4.5 mg/mL, respectively and kept frozen until analysis. Calibration curves for these two compounds were constructed based on seven concentration points (from 800 to 3.9 µg/mL).

### 3.7. Data Presentation and Statistical Analysis

For data analysis, the results of the conventional solvents’ extracts were converted to mg of initial plant dry biomass per mL of used solvent, based on the extraction yield, to allow for the comparison with the NADES results. The EC_50_ values were calculated for the ethanol extracts at a starting concentration of 4.23 mg of initial dry plant biomass/mL, for the acetone extract at 20 mg of initial plant dry biomass/mL, and for the NADES extracts at 25 mg of initial dry plant biomass/mL. EC_50_ values were calculated by sigmoidal fitting of the data using GraphPad Prism for Windows v.8.4.0 (GraphPad Software, La Jolla California). The area under the curve for the ORAC assay was calculated using the same software. Differences between means were analyzed by One-way ANOVA followed by Duncan’s new multiple range test (*p* < 0.05), using IBM SPSS Statistics for Windows v.25.0 (Armonk, New York: IBM Corp).

The LC-MS data in the form of the generated bucket table consisting of Rt:m/z pairs and the respective compound intensity were exported and uploaded to the MetaboAnalyst 5.0 [58] freeware (https://www.metaboanalyst.ca/) to estimate the missing values and to filter and normalize data (normalization by median). No transformation was generalized and the data matrix were mean-centered and divided by the square root of the standard deviation of each variable (Pareto scaling). The PCA score plot was used to present a natural correlation between the observations. To identify compounds contributing to differences between chromatographic profiles of ethanol, acetone, and NADES, we used the PLS-DA model with variable importance in projection (VIP) values (VIP ≥ 1.0) and *p* (corr) ≥ 0.5.

## 4. Conclusions

For NADES optimization, those consisting of ChCl with sucrose (1:2 with 40% of water), and fructose (1:2, 30% of water) exhibited the best stability. The extract made with ChCl and fructose always exhibited stronger antioxidant properties than the acetone extract. The ethanol extracts exhibited higher antioxidant properties, but their CCA was comparable to the NADES containing ChCl and sucrose. The phenolic profile was less complex in the NADES extracts with the absence of gallic acid glucoside, but other major antioxidants, such as catechins as well as myricetin and quercetin glycosides were present in comparable proportions in all the solvents. To the best of our knowledge, this is the first report on the use of NADES to extract antioxidant compounds from halophyte plants, and our results suggest that those combining ChCl, sucrose, and fructose (molar ratio 1:2) could replace acetone and ethanol in the extraction of antioxidants from sea knotgrass.

## Figures and Tables

**Table 1:** List of natural deep eutectic solvents (NADES) prepared in this work and the corresponding molar ratio of hydrogen bond acceptor (HBA) and hydrogen bond donor (HBD), water content, as well as stability, time, and temperature of the synthesis.

HBA:HBD	Molar Ratio	Water Content	Stability *	Time/Temperature of Synthesis
ChCl:Fru	1:2	30%	Stable	15 min/50 °C
ChCl:Gluc	1:2	30%	Unstable	60 min/55 °C
ChCl:Xyl	1:2	30%	Unstable	55 min/55 °C
ChCl:Suc	1:2	40%	Stable	50 min/55 °C

HBA: Hydrogen bond acceptor; HBD: Hydrogen bond donor; Ch: Choline; Ch: Chloride; Suc: Sucrose; Fru: Fructose; Gluc: Glucose. * NADES were considered stable if no significant crystallization was observed after 7 days of storage in the dark at room temperature.

**Table 2:** LC-ESI-qTOF-MS analysis of the extracts from *Polygonum maritimum* L. (sea knotgrass) with annotated peaks and semi-quantitative determination * of selected phenolic compounds. Identification was based on our previous reports on sea knotgrass [26,27,28,29].

No.	Compounds	t_R_	UV	*m/z* [M − H]^−^	Formula	MS^2^ Main-Ion	MS^2^ Fragments	Ethanol	Acetone	ChCl:Sucrose	ChCl:Fructose
1	6-O-Galloyl-glucose	0.9	272	331.0673	C_13_H_16_O_10_	169.0110	271,211,151	82.14	113.515		
2	Sucrose	1.0		341.1088, 387.1149 [M + FA]	C_12_H_22_O_11_	179.0519	161				
3	Fructose	1.0		179.0545, 225.0602 [M + FA]	C_6_H_12_O_6_	161.0421	149				
4	Citric acid *	1.2	209,272	191.0181	C_6_H_8_O_7_	147.0322	127	0.58	0.12	1.107	0.95
5	Gallate 3-glucoside^E^	1.3	272	331.0667	C_13_H_16_O_10_	169.0120	271,211,151,125	0.05	0.965		
6	Gallic acid^E^	1.4	213	169.0137	C_7_H_6_O_5_	125.0236		3.59	3.240	0.120	5.62
7	Acetyl-dihydroxyphenyl-D-glucopyranoside^E^	1.5	275	329.0869	C_14_H_18_O_9_	167.0328	152,283		0.182	0.333	
8	Epigallocatechin^E^	1.9	277	305.0661	C_15_H_14_O_7_	167.0326	219,179,261,237	2.30	5.919	0.365	0.84
9	Methylgallic acid-O-sulphate^E^	2.5	257	262.9864	C_8_H_8_O_8_S	183.0289				3.17	2.16
10	Catechin^E^	4.4	275	289.0704	C_15_H_14_O_6_	245.0798	203,221,151,137,125	0.84	2.336	0.09	
11	D-threo-Hexitol^E^	4.8	276	293.1232	C_12_H_22_O_8_	131.0691			0.134		0.25
12	Epicatechin^E^	5.2	275	289.0706	C_15_H_14_O_6_	245.0805	203,151,137,125	0.91	0.840	0.593	0.66
13	Caffeic acid 3-sulfate^E^	5.6	306	258.9906	C_9_H_8_O_7_S	179.0330	135	1.21	0.881	0.236	0.14
14	*p*-Coumaric acid-glucoside^E^	5.8	279	325.0932	C_15_H_18_O_8_	163.0376	119	1.18	1.001	0.570	1.19
15	Epigallocatechin-epicatechin 3-O-gallate^E^	7.4	277	745.1413	C_37_H_30_O_17_	305.0652	423,161,125	1.58	1.178	1.251	0.15
16	Coumaroylquinic acid^E^	7.6	275	337.0933	C_16_H_18_O_8_	191.0540	173,163		0.422		
17	Coumaroylquinic acid^E^	7.8	275	337.0921	C_16_H_18_O_8_	191.0542	173,163		0.947	0.434	0.25
18	Epigallocatechin gallate^E^	7.9	275	457.0776	C_22_H_18_O_11_	169.0117	305,125	0.86	0.417	0.210	0.22
19	Gallocatechin gallate^E^	8.2	276	457.0779	C_22_H_18_O_11_	169.0120	305,125	0.68	1.285	1.273	0.44
20	Ethylgallate sulfate^E^	8.4	277	277.0010	C_9_H_10_O_8_S	197.0436	182	0.31	0.430	0.620	
21	Ent-Epicatechin-ent-epicatechin 3-gallate^E^	8.5	274	729.1454	C_37_H_30_O_16_	289.0708	269,125,407,169,433		0.428		
22	Myricetin-galloylglucoside^F^	8.7	273	631.0944	C_28_H_24_O_17_	479.0826	316,169	1.49	1.174	0.513	0.30
23	Dihydroferulic acid 4-O-glucuronide^E^	8.9		371.0977	C_16_H_20_O_10_	249.0596	175,121		0.180		0.18
24	Ent-Epicatechin-ent-epicatechin^E^	9.2		577.1349	C_30_H_26_O_12_	289.0690	407,125,161,245,381	0.55	0.443	0.341	0.23
25	Ent-Epicatechin-ent-epicatechin 3-gallate^E^	9.3		729.1457	C_37_H_30_O_16_	289.0708	269,125,407,169,433	2.00	2.684	0.983	
26	Myricetin 3′-glucoside^F^	9.5		479.0833	C_21_H_20_O_13_	316.0221		1.43	0.675		0.39
27	Quercetin 3-(2′′-galloylglucoside)^F^	9.9		615.0994	C_28_H_24_O_16_	463.0878	300,313,271,241,169	2.01	3.744	0.736	0.32
28	Nepetin 4′-glucoside^F^	10.0		477.1036	C_22_H_22_O_12_	313.0553	433,169,163	0.27	0.357		0.16
29	Gossypetin 8-O-glucoside^F^	10.2		479.0832	C_21_H_20_O_13_	317.0290	169	2.38	5.326	0.807	0.32
30	Myricetin 3-O-rhamnoside^F^	10.4	218,263,351	463.0889	C_21_H_20_O_12_	316.0230	300	62.71	96.190	23.852	18.71
31	Epicatechin-3-gallate^E^	10.5	262, 354	441.0844	C_22_H_18_O_10_	169.0124	289,245,125				
32	Quercetin-3-O-galactoside	10.6	262, 355	463.0885	C_21_H_20_O_12_	300.0276		12.26	24.270	3.234	3.66
33	Quercetin-3-O-glucoside^F^	10.8	265, 352	463.0891	C_21_H_20_O_12_	300.0275		5.74	10.412	2.398	1.96
34	Catechin 3-O-rutinoside^E^	11.1	278	597.1838	C_27_H_34_O_15_	357.0983	387,417,315,459,239			0.01	0.07
35	NI	11.2		449.2036	C_27_H_30_O_6_	269.1387	209				0.13
36	Quercetin 7-xyloside^F^	11.4	274	433.0784	C_20_H_18_O_11_	300.0270		0.68	1.408		0.16
37	Rhamnetin 3-galactoside^F^	11.8	275, 351	477.1042	C_22_H_22_O_12_	315.0494	299,462	0.43	0.596	1.470	
38	Quercitrin^F^	11.9	272, 348	447.0937	C_21_H_20_O_11_	300.0272	284,255	3.91	7.597	0.343	0.94
39	NI	12.3		415.1991		161.0418	179	0.61	0.843	0.237	0.40
40	Myricetin^F^	12.5	276	317.0302	C_15_H_10_O_8_	178.9978	151,137,287	0.31	0.842	0.310	0.01
41	NI	12.7		583.1100		300.0269	463				
42	NI	12.8		471.0569	C_22_H_16_O_12_	193.0127	301,319,178,257	0.11			
43	Myricetin O-glucopyranoside^F^	12.9	277	479.0827	C_21_H_20_O_13_	317.0296		0.13	0.290		
44	Dihydroxyflavanone-sulfate^F^	13.2		367.0148	C_15_H_12_O_9_S	287.0548	151,135	0.29	0.140		
45	NI	13.4		347.0764	C_17_H_16_O_8_	165.0169	211,137				
46	Hydroxyflavanone-sulfate^F^	13.5		351.0160	C_15_H_12_O_8_S	271.0600	151	0.10	0.137		
47	Trihydroxyflavanone-sulfate^F^	13.6		383.0075	C_15_H_12_O_10_S	303.0503	151	0.07	0.139		
48	Quercetin^F^	14.9	369	301.0347	C_15_H_10_O_7_	151.0012	179,273,255,229	0.19	0.281		
49	Isorhamnetin^F^	15.6		315.0503	C_16_H_12_O_7_	300.0272	271,255	0.09	0.328		
50	Dihydroxy-8-oxooctadec-12-enoate	17.1		327.2176	C_18_H_32_O_5_	211.1332	229,291,171				
51	Trihydroxy-9-octadecenoic acid	18.1		329.2329	C_18_H_34_O_5_	211.1327	229,283,311,171				

The content mg/g extract and dry weight (dw) are expressed as epicatechin (E) and isoquercitrin (F) equivalents, respectively; NI: Not identified.

## Data Availability

The dataset is available upon request from the corresponding author.

## Sample Availability

Samples of extracts are available from the authors.
